# A Case of Biphasic Pulmonary Blastoma Treated with Carboplatin and Paclitaxel plus Bevacizumab

**DOI:** 10.1155/2015/842621

**Published:** 2015-05-13

**Authors:** Shinya Sakata, Sho Saeki, Sayuri Hirooka, Susumu Hirosako, Hidenori Ichiyasu, Hirotsugu Kohrogi

**Affiliations:** Department of Respiratory Medicine, Kumamoto University Hospital, 1-1-1 Honjo, Chuo-ku, Kumamoto 860-8556, Japan

## Abstract

*Background*. Pulmonary blastoma is a rare lung tumor similar to fetal lung tissues. Surgical resection at early stage is more curative than other treatments, but there is no standard treatment in unresectable cases. We show a case treated with carboplatin and paclitaxel plus bevacizumab. *Case*. A 68-year-old man received surgical resection and was diagnosed with biphasic pulmonary blastoma (pT3N0M0 stage IIB). Metastasis to the spleen was detected six weeks after the surgery. Carboplatin, paclitaxel, and bevacizumab were administered and showed an effect on the metastasis. Four courses of the chemotherapy were completed, but a metastasis was found and the metastatic tumor in the spleen was enlarged. After that, chemotherapy was not effective afterward and he died of the progression of biphasic pulmonary blastoma on the 292nd day of illness. *Conclusion*. In this case, chemotherapy with carboplatin and paclitaxel plus bevacizumab was temporarily efficacious for biphasic pulmonary blastoma.

## 1. Introduction

Pulmonary blastoma is a rare lung tumor similar to fetal lung tissues and is considered to be 0.25–0.5% of the primary pulmonary malignant tumor [1]. Surgical resection at early stage of pulmonary blastoma is more curative than other treatments, but its 5-year survival rate is 25% in stage I. At present, in unresectable cases chemotherapy and radiotherapy do not statistically reach the establishment of the standard treatment. Biphasic pulmonary blastoma, a subgroup of pulmonary blastoma, is characterized by histopathological heterogeneity intermixing of epithelial and mesenchymal malignant cells [2]. We show a case of biphasic pulmonary blastoma effectively treated with carboplatin and paclitaxel plus bevacizumab.

## 2. Case Report

**Figure 1:**
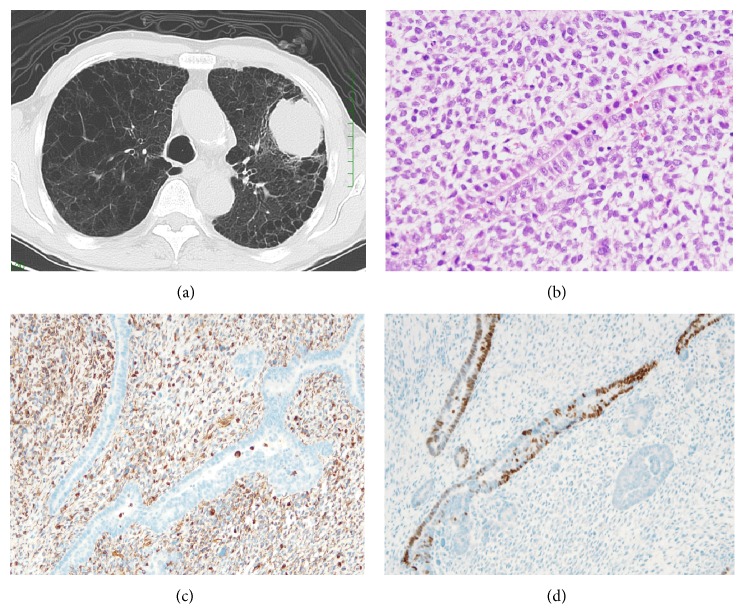
(a) Chest CT showing the mass opacity of the diameter 55 × 45 mm in size in the left upper lobe. Severe emphysema is visible in the background. (b) Pathology (hematoxylin and eosin staining, 400x) shows a part of carcinoma with slit-formed duct formation of carcinoma and immature mesenchymal cells. (c) Vimentin (100x) is diffusely positive for the mesenchymal cells. (d) TTF-1 (100x) is positive in parts of carcinoma with the slit-formed duct formation.

A 68-year-old man was admitted to our hospital because of a mass lesion (55 × 45 mm) in the left upper lobe of the lung on chest radiograph and computed tomography (CT) scan (Figure 1(a)).

Physical examinations were normal, and laboratory tests showed an elevated level of serum carcinoembryonic antigen (CEA) level (4.7 ng/mL). Ultrasound-guided biopsy of the tumor was performed. Pathology of the tissue stained with hematoxylin and eosin (H&E) staining showed malignant cells which were suspected large cell neuroendocrine carcinoma of the lung. Fluorodeoxyglucose-positron-emission tomography (FDG-PET) and brain magnetic resonance imaging (MRI) showed no apparent metastasis, and the clinical stage was cT3N0M0 stage IIB. Then, left upper lobectomy and lymph node dissection were performed.

The pathology of the surgically resected tumor with H&E staining demonstrated mixed epithelial and mesenchymal malignancies, biphasic pulmonary blastoma showing slit-formed duct formation of carcinoma, and immature mesenchymal cells (Figure 1(b)). Immunohistochemical staining for epithelial markers, CAM5.2 and TTF-1, was positive in epithelial areas (Figure 1(d)). The mesenchymal markers, vimentin and desmin, were positive in the mesenchymal areas (Figure 1(c)). These findings confirmed the diagnosis of biphasic pulmonary blastoma.

**Figure 2:**
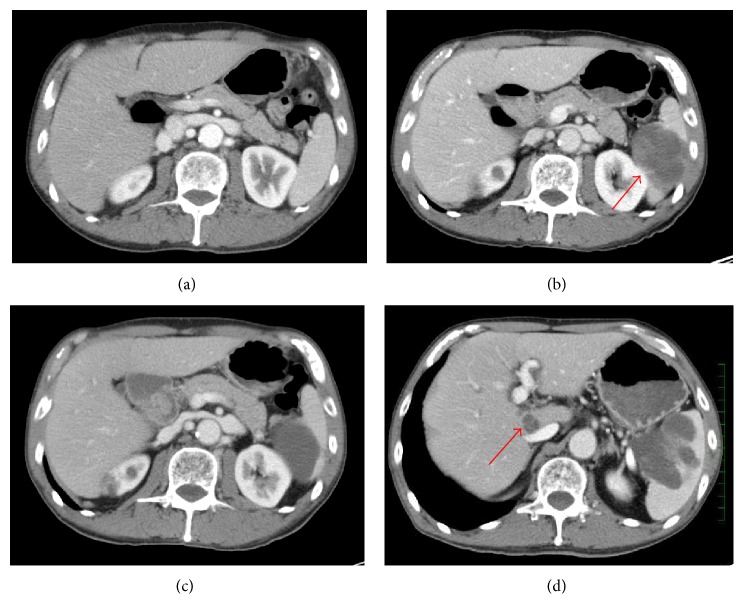
(a) There are no abnormal findings in abdominal CT scan before surgery. (b) Abdominal CT scan with contrast 6 weeks after surgery shows a mass with inhomogeneous opacity in the spleen (arrow). (c) Abdominal CT scan with contrast after the 3 courses of chemotherapy by carboplatin and paclitaxel plus bevacizumab. The tumor size is reduced and its inhomogeneous opacity changed to homogeneous opacity. (d) Abdominal CT scan with contrast after the 4 courses of chemotherapy shows a new metastasis to the liver and enlarged metastatic tumor in the spleen (arrow).

**Figure 3:**
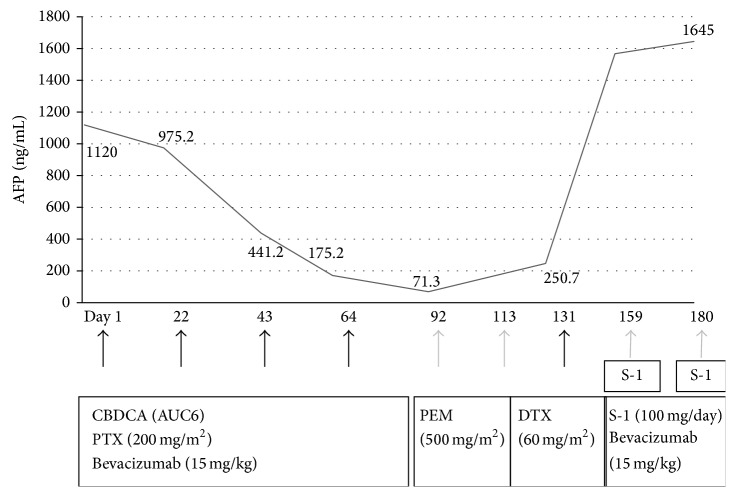
Clinical course showing decreased levels of AFP after the serial courses of chemotherapy with carboplatin and paclitaxel plus bevacizumab. After the recurrence, levels of AFP rapidly increased regardless of latter treatment with pemetrexed (PEM), docetaxel (DTX), and S-1 plus bevacizumab.

When he was admitted to our hospital to receive adjuvant chemotherapy six weeks after the surgery, abdominal CT showed a metastasis to the spleen (Figures 2(a) and 2(b)). Carboplatin area under the curve 6 and paclitaxel 200 mg/m^2^ plus bevacizumab 15 mg/kg on day 1 every 3 weeks were administered and the effect measurement in CT after two courses of the chemotherapy showed reduction of the metastatic lesion and its inhomogeneous opacity changed to homogeneous opacity (Figure 2(c)), and levels of tumor markers were decreased (Figure 3). After two courses of the chemotherapy, serum levels of AFP decreased from 1120.0 to 441.2 ng/mL, those of NSE decreased from 15.2 to 9.6 ng/mL, and those of CEA decreased from 4.7 to 3.1 ng/mL. Four courses of the chemotherapy were completed, but a new liver metastasis was found and the metastatic tumor in the spleen was enlarged (Figure 2(d)). On the 292nd day of illness he died of the progression of pulmonary blastoma without showing a response to pemetrexed 500 mg/m^2^ on day 1 every 3 weeks, docetaxel 60 mg/m^2^ on day 1 every 3 weeks, and S-1 80 mg/m^2^/day on days 1 to 14 plus bevacizumab 15 mg/kg on day 1 every 3 weeks as the latter treatment (Figure 3). Autopsy showed multiple metastases to the spleen, liver, pleura, rib, stomach, duodenum, heart, and cervical lymph nodes. The cut surface of metastatic tumors showed significant necrosis and hemorrhage.

## 3. Discussion

Pulmonary blastoma is classified into three subgroups: (1) biphasic pulmonary blastoma, characterized by histopathological heterogeneity intermixing of epithelial and mesenchymal malignant cells, (2) well-differentiated fetus adenocarcinoma, characterized by a monophasic epithelial tumor, and (3) pleuropulmonary blastoma, characterized by a monophasic mesenchymal tumor. However, according to World Health Organization (WHO) classification of lung tumors published in 1999, well-differentiated fetus adenocarcinoma is classified into a histological variant of adenocarcinoma, and pleuropulmonary blastoma is classified into soft tissue tumors [2].

Pulmonary blastoma is a rare lung tumor similar to fetal lung tissues and is considered to be 0.25–0.5% of the primary pulmonary malignant tumor [1]. The mean tumor diameter is known to be 101 mm (20–270 mm) [3]. In the present case, tumor developed in peripheral area, and the diameter 55 mm at the initial diagnosis showed a rapid growth to 80 mm in three weeks at the surgical resection. Serum levels of tumor markers, AFP, NSE, and CEA, were increased as shown in the previous reports [4]. In particular, AFP positive tumor cells are seen in many pulmonary blastoma cases [5]. Therefore, an elevated level of AFP in a patient with lung tumor is one of suggestions to suspect pulmonary blastoma. Because it is difficult to obtain tissues containing both epithelial and mesenchymal tumor by transbronchial biopsy, its pathological diagnosis is done by tissues obtained by surgical resection in most cases of pulmonary blastoma. In fact, the present case was preoperatively diagnosed as large cell neuroendocrine carcinoma by percutaneous needle biopsy.

The prognosis of biphasic pulmonary blastoma is extremely poor. The size of more than 50 mm is a significant predictor of a poor prognosis [3]. In the present case, the tumor was 80 mm in diameter at the surgical resection. Although surgical resection at early stage of pulmonary blastoma is more curative than any other treatment [3], its 5-year survival rate is 25% in stage I. At present, in unresectable cases, chemotherapy and radiotherapy do not statistically reach the establishment of the standard treatment.

One case showed partial response to ifosfamide plus doxorubicin with radiotherapy for a postoperative recurrence [6]. In another case, cisplatin plus etoposide with radiotherapy made surgical resection possible for postoperative recurrence. Also, a case of pulmonary blastoma responding to sorafenib was reported [7].

The present case showed recurrence with the distant metastasis six weeks after the surgery. Because pulmonary blastoma was nonsquamous cell carcinoma, we selected the regimen carboplatin and paclitaxel plus bevacizumab as one of the standard treatments. Bevacizumab is the IgG1 hominization monoclonal antibody targeting the vascular endothelial growth factor (VEGF), and it inhibits neovascularization by binding to blood VEGF inhibiting the bond with the VEGF receptor of vascular endothelial cells and shows antitumor effect [8].

In the present case, the treatment after the 3 courses showed decreased levels of AFP and decreased tumor size of the metastasis, suggesting that this regimen is effective on biphasic pulmonary blastoma. Further accumulation of knowledge and experience is needed to establish effective treatment (including chemotherapy) of biphasic pulmonary blastoma.

## 4. Conclusion

To our knowledge, there is no report of responders to bevacizumab combination regimen for the biphasic pulmonary blastoma so far. The present case is valuable to establish future chemotherapy for biphasic pulmonary blastoma. Accumulation of the future cases is expected.
